# Patient Profile and Referral Pattern in a Tertiary Care Infectious Disease Hospital of Bangladesh: A Cross-Sectional Study

**DOI:** 10.7759/cureus.112037

**Published:** 2026-07-04

**Authors:** Mohammad Jahid Hasan, Mohammad Mehedi Hasan Rabbi, Abdullah Al Tahsin Chowdhury, Zubayer Hasan, Ashish Paul, Ashrak Shad Pyash, Homayra Rahman Shoshi, Aninidita Das Barshan, Mohammad Abdullah Saeed Khan

**Affiliations:** 1 Epidemiology and Public Health, Pi Research Development Center, Dhaka, BGD; 2 Laboratory, Dhaka Medical College Hospital, Dhaka, BGD; 3 Laboratory, Mugda Medical College, Dhaka, BGD; 4 Laboratory, Bangladesh Assisted Conception Centre Women's and Children Hospital, Dhaka, BGD; 5 Intensive Care Medicine, Monowara Hospital (Pvt.) Ltd., Dhaka, BGD; 6 Public Health, State University of Bangladesh, Dhaka, BGD; 7 Medicine, Dhaka Medical College Hospital, Dhaka, BGD; 8 Public Health, Infectious Disease Hospital, Dhaka, BGD

**Keywords:** bangladesh, infectious diseases, patient profile, public healthcare system, referral pattern, self-referral, tertiary care hospital

## Abstract

Introduction

Infectious diseases are attributable to a large proportion of the disease burden in Bangladesh. The disease profile and referral pattern of these patients are important for understanding healthcare utilization and improving system efficiency in resource-limited settings. Hence, the objective of the present study was to assess the patient profiles and referral patterns at the Infectious Diseases Hospital (IDH), Dhaka, Bangladesh.

Methods

This cross-sectional study was conducted at IDH, Mohakhali, Bangladesh. Using a systematic sampling, a total of 308 patients (161 outpatients and 147 inpatients) were recruited. Data regarding patients’ sociodemographic, diagnosis, and referral were collected through face-to-face interviews using a semi-structured questionnaire. Descriptive statistics were used to represent the findings.

Results

The mean age of the patients was 31.5 years (standard deviation, SD 16), with 66% (n=204) being male subjects and 56% (n=172) residing in urban areas. Among outpatients, 93% (n=150) presented with animal bites or scratches, while inpatients presented with more severe conditions, including contagious viral infections (28.6%, n=42), tetanus (26%, n=38), and acquired immune deficiency syndrome (AIDS) (25%, n=37). Overall, 39.6% (n=122) of patients were institutionally referred, with higher referral rates among inpatients (68%, n=100) than outpatients (13.7%, n=22). Primary care facilities accounted for 33% (n=40) of institutional referrals. Inadequate treatment facilities (54%, n=54), shortages of specialist physicians and other staff (41%, n=41), insufficient diagnostic facilities (33%, n=33), and inadequate vaccine or medicine supply (22%, n=22) were the most common reasons for both institutional and self-referral.

Conclusion

A substantial number of patients were self-referred at IDH. Strengthened primary care infrastructure and referral systems would optimize tertiary care utilization and improve healthcare access in Bangladesh.

## Introduction

An effective referral system is essential for ensuring efficient and equitable healthcare delivery [1,2]. It facilitates the seamless transfer of patients across different levels of care, enabling them to access the right services at the right time. In low- and middle-income countries (LMICs), however, referral systems often fail to function as intended due to resource limitations, structural inefficiencies, and the lack of awareness among patients and healthcare providers [1]. Bangladesh, like many LMICs, faces significant challenges in implementing an effective referral system, particularly within its public healthcare framework [3].

The healthcare delivery system of Bangladesh is structured into three tiers: primary, secondary, and tertiary care. Primary care services are provided by several health facilities, such as community clinics at the village level, union health sub-centers, and upazila health complexes at the sub-district level, which offer basic healthcare services to rural and semi-urban populations. Secondary care is delivered through district hospitals, and tertiary care is mostly delivered by medical colleges and super-specialized hospitals, mostly situated in the capital city of Dhaka and other regional cities [4,5]. This hierarchical system is designed to ensure that patients with less complex conditions receive care at the primary or secondary levels, while those with more serious conditions are referred to tertiary facilities. This structured approach would not only optimize resource utilization but also ensure that specialized care at tertiary hospitals is reserved for those who truly need it. However, the reality is starkly different. A substantial proportion of the patients bypass primary and secondary care facilities altogether, directly seeking treatment at tertiary hospitals [3]. This results in overburdened healthcare providers, strained infrastructure, and compromised quality of care at tertiary care facilities.

Bangladesh is experiencing an epidemiological transition, struggling with the dual burden of communicable and non-communicable diseases [6,7]. Despite a rising prevalence of non-communicable conditions, infectious diseases continue to represent a substantial portion of the country’s disease burden [7]. Infectious diseases such as tuberculosis (TB), malaria, chickenpox, measles, tetanus, etc. have long been prevalent in Bangladesh [8-10]. These diseases are now joined by emerging and re-emerging infections like acquired immunodeficiency syndrome (AIDS) and emerging respiratory viral diseases, pressing the country’s already strained healthcare infrastructure [11,12].

To address the burden of infectious diseases requiring specialized management and isolation, such as tetanus, rabies, chickenpox, and highly transmissible respiratory viruses, Bangladesh has an established network of infectious disease hospitals at both the divisional and national levels. At the divisional level, these hospitals are typically attached to the regional medical college hospitals. However, the national-level Infectious Disease Hospital (IDH) situated in the capital city of Dhaka, operating independently under the Directorate General of Health Services (DGHS), functions as a tertiary referral center for complex and severe infectious disease cases across the country [4]. This hospital is uniquely positioned within the healthcare system of Bangladesh, offering specialized care that includes isolation facilities, advanced diagnostics, and expert management for conditions like tetanus, rabies, AIDS, and emerging respiratory infections. Understanding the disease patterns of patients utilizing services of this hospital could shed light on the broader infectious disease trends within the country. Furthermore, as with other tertiary care facilities in Bangladesh, this hospital is also likely to be strained by the influx of self-referred cases that may not require specialized care [3]. Exploring the referral patterns of these patients might identify the policy shortcomings and implement strategies to redirect non-severe cases to primary or secondary care facilities, thereby optimizing resource allocation and alleviating the burden on tertiary care services of this hospital. Hence, the objective of the present study was to assess the patient profile and their referral patterns at IDH.

## Materials and methods

Study design and place

This cross-sectional study was conducted at the IDH, Dhaka, Bangladesh, over a period of six months from July 1 to December 31, 2022. IDH is a 100-bed specialized tertiary care hospital dedicated to infectious diseases. It provides both specialized inpatient and outpatient services and serves as a major referral center for infectious disease management in Bangladesh.

Participants

The study population comprised all inpatients and outpatients at IDH. As our outcome variable was the referral pattern of patients attending the hospital, and institutional referral is the major mode of referral, the sample size was estimated using the single-population proportion formula using the institutional referral rate: \[n = \frac{z^2 \times p \times (1 - p)}{d^2}\] where z is the standard normal deviate (1.96 for 95% confidence level), p is the estimated proportion, and d is the margin of error. In the absence of prior data on referral patterns in infectious disease hospitals, a referral rate of 41% reported from a tertiary care hospital in Bangladesh [3] was used to obtain the minimum required sample size. From this formula, the required sample size was 372 participants. As we recruited patients from both the inpatient and outpatient departments of the hospital, an equal allocation of 186 from each department was considered to ensure adequate representation of both patient groups and enable comparison of their referral characteristics.

Eligibility criteria included pediatric and adult patients with a confirmed diagnosis of infectious diseases, as defined by the International Classification of Diseases, Tenth Revision (ICD-10) [13], and/or those with animal bites or scratches requiring rabies vaccination. Patients with non-infectious diagnoses or those who were severely ill and unable to participate in interviews (along with their attendants) were excluded.

A systematic sampling method was used for the recruitment of the patients in the present study. According to the hospital records, approximately 200 patients visited the outpatient department daily, and 50 patients were admitted as inpatients during the preceding month of the study conducted. Based on a 30-day data collection period, the required daily recruitment was six patients, evenly distributed between the inpatient and outpatient departments. This led to a sampling interval of 31 for the outpatient department and nine for the inpatient department, ensuring a representative sample. Every 31st patient from the outpatient and every ninth patient from the inpatient departments were recruited.

Data collection

Face-to-face interviews were conducted for data collection, either with patients or with their attendants, if patients were unable to respond. Outpatient interviews were conducted during consultations, while inpatient interviews were completed within 24 hours of admission by the attending physicians. A semi-structured questionnaire was used, consisting of three sections: (i) sociodemographic information: this section collected patient details such as age, gender, employment status, place of residence, and approximate distance from the hospital; (ii) medical information: data on patients’ diagnoses were recorded; and (iii) referral-related information: questions assessed patients’ awareness of the referral system (defined as awareness of the question, “have you heard about the referral system in healthcare?”), the referral method (self-referral vs. institutional referral), the referring hospital (if institutional referral), and reasons for referral.

The questionnaire was developed by a panel of experts based on prior literature and their experience with the healthcare system of Bangladesh (appendix). The initial draft was revised after thorough discussions with the co-investigators. After that, a Bangla version was prepared using back-translation process performed by two independent translators. To ensure clarity and reliability, the questionnaire was then pre-tested on patients from Dhaka Medical College Hospital (n=30), who were not included in the final analysis, leading to refinements for linguistic precision.

Ethics statement

The study protocol was approved by the Ethical Review Committee of the Dhaka Medical College (approval no: ERC-DMC/ECC/2020/78), and formal permission was obtained from the IDH authority. Written informed consent was obtained from all participants before enrollment. In cases where patients were unable to consent, their guardians provided consent on their behalf. The study adhered to the ethical principles outlined in the Declaration of Helsinki.

Statistical analysis

Data analysis was performed using Stata version 17.0 (StataCorp LLC, College Station, TX, US). Descriptive statistics were used to summarize the findings. Continuous variables were presented as means with standard deviations (SD), while categorical variables were expressed as frequencies and percentages. Referral-related data were visualized using a schematic diagram to depict patients’ pathways to IDH. Comparisons between groups were made using the chi-square test for categorical variables and the independent t-test for continuous variables, as appropriate. Statistical significance was set at a two-sided p-value of <0.05.

## Results

Sociodemographic characteristics

A total of 308 patients were included, of whom 147 (47.7%) were from the inpatient department, and 161 (52.3%) were from the outpatient department. The mean age of patients was 31.5 years (SD 16), with the majority (56%, n=174) falling within the 18-to-39-year age group. Nearly two-thirds (66%, n=204) were male subjects, and 60% (n=186) reported being employed. Over half (56%, n=172) of the patients resided in urban areas, and 67% (n=206) lived within 20 km of the hospital. Knowledge about the referral system was reported by 78% (n=239) of the patients. Differences between inpatient and outpatient groups were not significant, except for residence and distance from the hospital. Most outpatient attendees were from nearby urban areas, whereas inpatients exhibited more geographic diversity (Table 1).

**Table 1:** Sociodemographic characteristics of the patients (n=308) *p-values were determined by independent samples t-test, Chi-squared test and Fisher’s exact test where appropriate; IDH: Infectious Disease Hospital.

Characteristic	Total (n=308)	Inpatient (n=147)	Outpatient (n=161)	p-value*
Age (years), mean (SD)	31.53 (16.09)	32.76 (17.20)	30.40 (14.97)	0.162
Age group (years), n (%)				0.305
<18	62 (20.13)	29 (19.73)	33 (20.50)	
18-29	78 (25.32)	33 (22.45)	45 (27.95)	
30-39	96 (31.17)	44 (29.93)	52 (32.30)	
40-49	39 (12.66)	25 (17.01)	14 (8.70)	
50-59	19 (6.17)	8 (5.44)	11 (6.83)	
≥60	14 (4.55)	8 (5.44)	6 (3.73)	
Sex, n (%)				0.263
Male	204 (66.23)	102 (69.39)	102 (63.35)	
Female	104 (33.77)	45 (30.61)	59 (36.65)	
Employment status, n (%)				
Employed	186 (60.39)	97 (65.99)	89 (55.28)	0.095
Not employed	122 (39.61)	50 (34.01)	72 (44.72)	
Residence, n (%)				<0.001
Rural	136 (44.16)	100 (68.03)	36 (22.36)	
Urban	172 (55.84)	47 (31.97)	125 (77.64)	
Distance from IHD, n (%)				0.003
<20 km	206 (66.88)	58 (39.46)	148 (91.92)	
>20 km	102 (33.12)	89 (60.54)	13 (8.08)	
Knowledge about referral system, n (%)				0.083
Yes	239 (77.59)	122 (82.99)	117 (72.67)	
No	69 (22.41)	25 (17.01)	44 (27.33)	

Patient diagnoses

The most frequently reported diagnosis was animal bite/scratch, accounting for 51.6% (n=159) of cases, followed by contagious viral infections (e.g., chickenpox, measles) at 14% (n=44), tetanus at 12% (n=38), AIDS at 12% (n=37), respiratory infections (e.g., tuberculosis, pneumonia) at 5% (n=16), parasitic diseases (e.g., malaria, filariasis, kala-azar) at 1.3% (n=4), and other infectious diseases at 3.2% (n=10).

The disease distribution varied significantly between inpatient and outpatient departments. Among outpatients, 93% (n=150) were diagnosed with animal bite/scratch, dominating the case profile. Conversely, among inpatients, the most common diagnoses were contagious viral infections (28.6%, n=42), tetanus (26%, n=38), HIV infection/AIDS (25%, n=37), and respiratory infections (9%, n=13) (Table 2).

**Table 2:** Diagnosis of the patients in the inpatient and outpatient departments (n=308) TB: tuberculosis.

Diagnosis category	Total (n=308)	Inpatient (n=147)	Outpatient (n=161)
Animal bite/scratch	159 (51.62)	9 (6.12)	150 (93.17)
Contagious viral infections (e.g., chickenpox, measles, etc.)	44 (14.29)	42 (28.57)	2 (1.24)
HIV infection/AIDS	37 (12.01)	37 (25.17)	0 (0.00)
Tetanus	38 (12.34)	38 (25.85)	0 (0.00)
Parasitic disease (Malaria, Filariasis, kala azar)	4 (1.30)	4 (2.72)	0 (0.00)
Respiratory infections (TB, Pneumonia, etc.)	16 (5.19)	13 (8.84)	3 (1.86)
Others	10 (3.25)	4 (2.72)	6 (3.73)

Referral patterns

Overall, 39.6% (n=122) of patients were referred by other healthcare facilities, while 60.4% (n=186) were self-referred. Institutional referral rates were significantly higher among inpatients (68%, n=100) than outpatients (13.7%, n=22). Among referred patients, primary care facilities were the most frequent referring facility (33%, n=40), followed by secondary care facilities (26%, n=32), other tertiary care facilities (25%, n=30), and private healthcare facilities (16%, n=20). Among the institution-referred patients, 62% (n=76) presented with a referral note, with higher rates among inpatients (69%, n=69) compared to outpatients (32%, n=7) (Table 3).

**Table 3:** Referral related characteristics of the patients (n=308)

Referral characteristic	Total (n=308)	Inpatient (n=147)	Outpatient (n=161)	p-value
Referral pattern				<0.001
Institutional referral	122 (39.61)	100 (68.03)	22 (13.66)	
Self-referral	186 (60.39)	47 (31.97)	139 (86.34)	
Referring center (n=122)				
Primary care facilities	40 (32.79)	32 (32.00)	8 (36.36)	0.362
Secondary care facilities	32 (26.23)	27 (27.00)	5 (22.73)	
Tertiary care facilities	30 (24.59)	24 (24.00)	6 (27.27)	
Private clinics/hospitals	20 (16.39)	17 (17.00)	3 (13.64)	
Referral note (n=122)				<0.001
Yes	76 (62.29)	69 (69.00)	7 (31.82)	
No	46 (37.71)	31 (31.00)	15 (68.18)	

A schematic diagram of patient pathways to IDH is presented inFigure 1.

**Figure 1:**
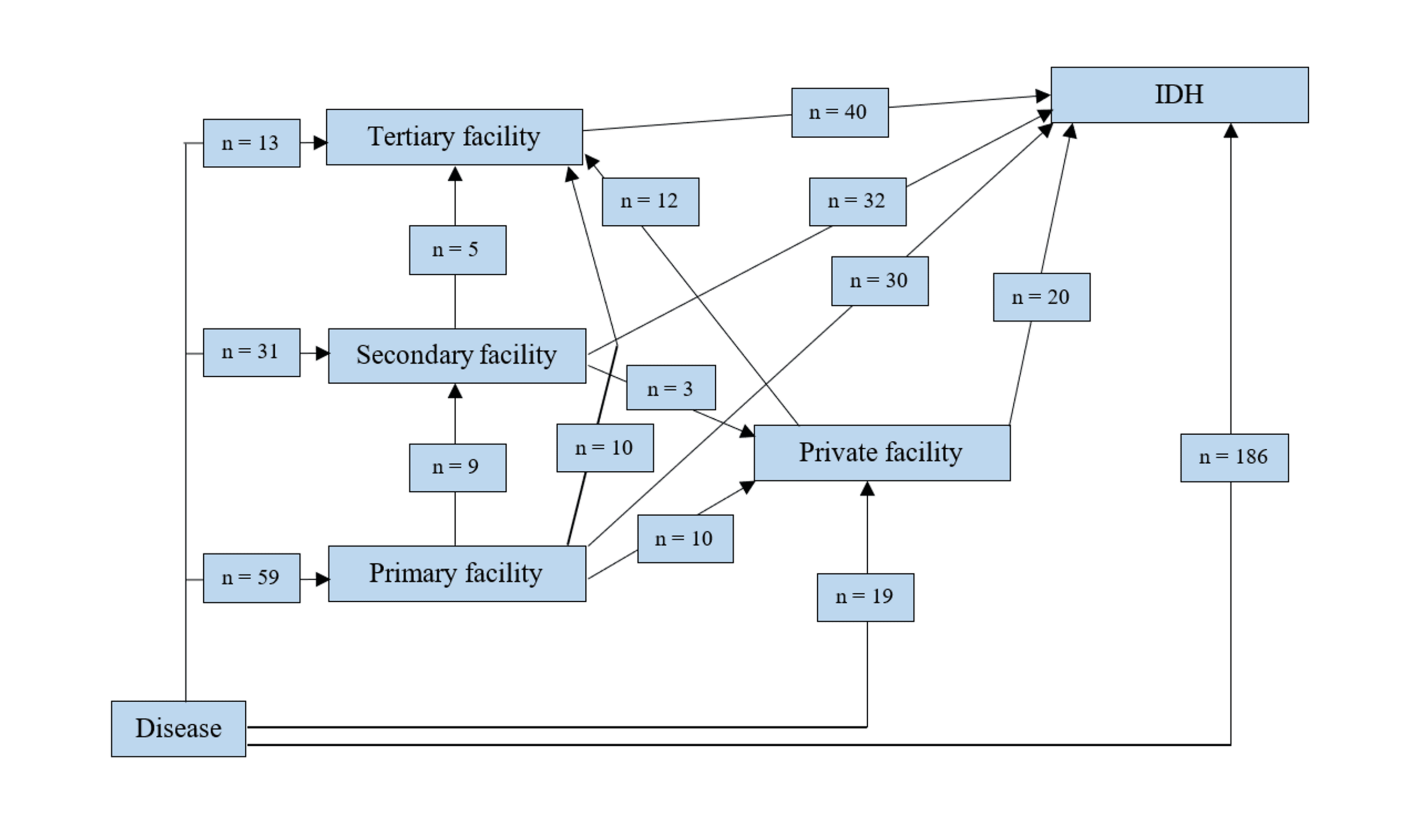
A schematic diagram of the various patient journeys to IDH (n=308) IDH: Infectious Disease Hospital. Image credit: Created by the authors using Microsoft Word (Microsoft Corp., Redmond, WA, USA).

Of the total 308 patients, 186 (60.4%) visited IDH directly by self-referral. Among the remaining patients, 59 (19.2%) initially attended primary care facilities, 31 (10.1%) attended secondary care facilities, 13 (4.2%) attended tertiary care facilities, and 19 (6.2%) visited private healthcare providers. From the 59 patients (19.2%) at primary care facilities, 30 (10%) were referred directly to IDH, while nine (2.9%) were referred to secondary care facilities and 10 (3.2%) to tertiary care facilities. An additional 10 patients (3.2%) visited private facilities from primary care. Among the 40 patients (13%) at secondary care facilities (31 initial attendees plus nine referred from primary care), 32 (10.4%) were referred directly to IDH, while five (1.6%) were referred to tertiary care facilities, and three (1%) visited private facilities. Of the 19 patients (6.2%) initially attending private facilities, 20 (6.5%) were referred directly to IDH, and 12 (3.9%) were referred to tertiary care facilities.

Self-referred patients were predominantly diagnosed with animal bite/scratch (71%, n=133), followed by AIDS (8.6%, n=16), contagious viral infections (7.5%, n=14), and tetanus (5%, n=9). In contrast, among facility-referred patients, the most common diagnoses were contagious viral infections (24.6%, n=30), tetanus (24%, n=29), and AIDS (17%, n=21).

Among facility-referred patients, the leading reasons for referral included inadequate treatment facilities, such as a lack of isolation wards (54%, n=54), shortages of specialist physicians and other staff (41%, n=41), insufficient diagnostic facilities (33%, n=33), and inadequate vaccine or medicine supply (22%, n=22). Perceived reasons for self-referral were dominated by similar inadequacies in treatment facilities (63%, n=63) and diagnostic facilities (31%, n=31). Other reasons included higher treatment costs at other facilities (22%, n=22) and previous referral to IDH for the same condition (21%, n=17).

## Discussion

Our study provided an overview of the sociodemographic profiles, disease patterns, and referral dynamics of the patients attending a tertiary care IDH in Bangladesh. We observed a predominance of male and younger age-group patients. Patients visiting the outpatient department were mostly presenting with animal bites or scratches, seeking post-exposure prophylaxis for rabies. Conversely, inpatients represented more severe cases, including contagious viral infections, tetanus, and AIDS. Approximately 40% of the patients were referred by other healthcare facilities, while the rest were self-referred. Among the referring facilities, primary care facilities had the highest frequency, followed by secondary and other tertiary care facilities. Inadequate facilities were the most commonly cited reason for both self-referral and institutional referral by the patients. 

We found a predominance of male patients falling in the younger age group, who were mostly employed, in both the inpatient and outpatient departments. Besides, the majority of patients visiting the outpatient department were from urban areas, while the geographic distribution of the patients admitted to the inpatient department was more evenly distributed between rural and urban areas. This male gender and working age group predominance was also reported by a study conducted among the patients with tetanus in the same hospital by Khan et al. [14]. Besides, sociocultural and economic factors might also lead to gender disparities in healthcare access, with men more likely to seek care compared to women due to societal norms and gender related behavior [15,16].

We also found that almost 60% of patients were self-referred to IDH, and the rest were referred from other lower-tier facilities. However, around one-third of those who were referred by other healthcare facilities visited the secondary or tertiary care facilities directly. Among institutional referrals, primary care facilities accounted for the highest proportion, yet these referrals often bypassed the secondary care facilities and directed patients straight to the IDH or other tertiary care facilities. There is a scarcity of evidence regarding the referral pattern in the context of the health system of Bangladesh. However, a recent study reported similar findings, where almost 59% of the patients in tertiary care hospitals were self-referred, and among the institutional referrals, almost 40% were directly from primary care facilities [3].

The rate of institutional referral in our study was higher among the inpatients compared to the outpatients, reflecting that more severe cases were referred from the health facilities. Their disease pattern also echoes this phenomenon, as diverse infectious diseases, including contagious viral infections, tetanus, and AIDS, were referred from other health facilities, while the outpatient visitors were mostly self-referred with complaints of animal bite or scratch and for receiving rabies post-exposure prophylaxis. An additional notable finding is that the majority of cases treated at IDH were uncommon infectious diseases, which required specialized management or isolation facilities, such as tetanus and AIDS, rather than more prevalent conditions like tuberculosis (TB), pneumonia, or malaria. This distribution can be attributed to the presence of robust national control programs for common infectious diseases, which enable their effective management at lower-tier healthcare facilities or general tertiary care hospitals [8,9,17].

Patients often cited insufficient diagnostic and treatment facilities, shortages of specialized physicians and other staff, inadequate supply of medicine and vaccines, and higher treatment cost as reasons for both self-referral and institutional referral from primary and secondary care facilities. These reasons of referral were also reported by the patients from other tertiary care facilities of the country [3], which indicates the inefficient referral system and resource constrained healthcare setting of the country.

We also observed over-utilization of tertiary care facilities like the IDH for routine services, such as post-exposure prophylaxis for rabies and other vaccination, which indicates the underutilization and under-resourcing of primary care facilities. This misallocation of services strains the capacity of tertiary centers, limiting their ability to manage severe and complex cases. Strengthening primary care services, especially in urban areas, is crucial to addressing these inefficiencies by improving accessibility, building capacity through investment in infrastructure and training, and establishing robust referral systems to ensure appropriate care at the right level [2]. Financial and geographic barriers also drive self-referral to IDH, with patients perceiving the center as more cost-effective. Expanding primary care services in underserved areas and implementing financial protection mechanisms could mitigate these challenges [18,19]. Aligning these reforms with the universal health coverage (UHC) mandate requires decentralizing services, ensuring adequate resource allocation, and building workforce capacity. Policy measures, including investments in primary care infrastructure, functional referral systems, community awareness campaigns, and regular evaluation of healthcare utilization, are essential [2,19]. These steps would optimize healthcare delivery, reduce patient burden on tertiary facilities, and improve access to equitable and efficient care across the system.

There are some limitations of our study. Firstly, the study was conducted at a single tertiary care infectious disease hospital in Dhaka city, limiting the generalizability of the findings to other regions or healthcare settings in Bangladesh. Secondly, the study focused on a specific subset of patients, predominantly those seeking care for infectious diseases, which may not fully represent the broader spectrum of referral patterns and disease profiles across the healthcare system. Thirdly, despite employing a systematic sampling approach, there is a potential for selection bias. Differences in healthcare-seeking behavior of patients who were self-referred and those who were referred by institutions could have influenced both their inclusion and the outcomes of the study. Fourthly, as a cross-sectional descriptive study, the findings are limited to associations and cannot establish causality. The study did not include multivariable adjustment for potential confounders, which may have influenced the observed referral patterns and patient characteristics. Finally, the sample size was slightly smaller than the calculated requirement, with 308 participants included instead of the intended 372, potentially reducing the statistical power of the study. Additionally, this study was quantitative in nature. Future qualitative research could complement these findings by exploring the underlying reasons of patients’ referral decisions.

## Conclusions

Most outpatients visiting the hospital sought medical care following animal bites or scratches, while inpatients more commonly presented with severe conditions such as contagious viral infections, tetanus, and AIDS. The majority of patients were self-referred and cited inadequate facilities at lower-tier centers as the main reason for attending the hospital. Strengthening primary care facilities and improving their diagnostic and management capacity could reduce patient burden at tertiary-level facilities. In addition, a structured referral pathway may enhance system efficiency.

## Appendices

**Table 4:** English questionnaire used in the study The researchers would state the following before administering the questionnaire: I would like to start by asking you some background questions before asking you questions on your health. This information is confidential and will only be used for research purposes.

Referral system in Bangladesh: Perception and reality		
Question no.	Questions	Possible response with instructions
Q.01	What is the name of the hospital?	______________________________
	Name of the department	
		Inpatient/Outpatient
Q.02	Record sex as observed	Male/Female
Q.03	How old are you?	
Q.04	What is the address of resident?	Village/area of house:…………………….. Thana/Upazila Municipality/city corporation District:
Q.05	What is the religion?	1. Islam
		2. Hindu
		3. Christian
		4. Buddhist
		5. Others (please specify) …………….
Q.06	What is your current job?	1. Government Employee
		2. Non-Government employee
		3. Self-employed
		4. Employer
		5. Not working for pay (If not working for pay: please answer Q.208)
Q.07	During the last 12 months, what has been your main occupation?	1. Legislator, Senior Official, or Manager
		2. Professional (engineer, doctor, teacher, clergy, etc.)
		3. Technician or Associate Professional (inspector, finance dealer, etc.)
		4. Clerk (secretary, cashier, etc.)
		5. Service or sales worker (cook, travel guide, shop salesperson, etc.)
		6. Agricultural or fishery worker (vegetable grower, livestock producer, etc.)
		7. Craft or trades worker (carpenter, painter, jewelry worker, butcher, etc.)
		8. Plant/machine operator or assembler (equipment assembler, sewing-machine operator, driver, etc.)
		9. Elementary worker (street food vendor, shoe cleaner, etc.)
		10. Armed forces (government military)
Q.08	What is the main reason you are not working for pay?	1. Homemaker / caring for family
		2. Looked but can’t find a job
		3. Doing unpaid work / voluntary activities
		4. Studies / training
		5. Retired / too old to work
		6. Ill health
		7. (please specify) …………….
Q.09	What is the highest level of education that you have completed?	1. No formal schooling
		2. Less than primary school
		3. Primary school completed
		4. Secondary school completed
		5. High school (or equivalent) completed
		6. College / pre-university / University completed
		7. Post graduate degree completed
Q.10	Diagnosis	
	Utilization of referral system (Source of referral)	
Q.11	Who advised you to come here (admit this hospital)?/OPD visit?	(1) Self: a. Yes b. No; If yes, please answer Q 212 and If No (Q. no:2-12), please answer Q 213; (2) Kabiraz: a. Yes b. No; (3) Palli doctor: a.Yes b.No; (4) Friends or family: a.Yes b.No; (5) Village doctor: a.Yes b.No; (6) Community clinic: a.Yes b.No; (7) Union subcenter: a.Yes b.No; (8) Upazilla Health complex: a.Yes b.No; (9) District hospital: a.Yes b.No; (10) Another tertiary hospital: a.Yes b.No; if yes, please specify……………………; (11) Private clinic: a.Yes b.No; (12) Others (please specify) ……………. Please try to make a flow chart about the referral centers used by patients: ………………………………
Q.12	Reasons for referring patients by self:	Response or comments done by attendant or care giver or by the patient: (More than one response could be possible: (1) Inadequate treatment: a.Yes b.No; If yes, please specify; (2) Inadequate facilities: a.Yes b.No; If yes, please specify ; (3) Inappropriate behavior: a.Yes b.No; If yes, please specify; (4) Higher cost: a.Yes b.No; If yes, please specify; (5) Patients condition deteriorating: a.Yes b.No; If yes, please specify; (6) Others: a.Yes b.No; If yes, please specify………………………………
Q.13	Reasons for referring patients by hospital facilities:	Response or comments done by physicians: (More than one response could be possible): (1) Difficult cases: a.Yes b.No; If yes, please specify; (2) Required additional management (e.g., chemotherapy, etc.) : a.Yes b.No; If yes, please specify; (3) Required additional equipment for diagnosis (e.g., CT scan, MRI, angiogram, etc.) : a.Yes b.No; If yes, please specify; (4) Lack of expert physicians (e.g , anesthetists for CS): a.Yes b.No; If yes, please specify; (5) Lack of appropriate facility (e.g., ICU, etc.) : a.Yes b.No; If yes, please specify; (6) When the illness has no solution (end-stage disease) : a.Yes b.No; If yes, please specify; (7) Others: : a.Yes b.No; If yes, please specify……………………………………….
Knowledge and perception of referral system of health care service		
Q.14	If hospital facilities referred the patients, have the patient received referral note?	1. a.Yes b.No; If No, please specify……………………………………….
Q.15	Do you think, it was a good decision to admit here??	1. a.Yes b.No; If yes, please specify……………………………………….
Q.16	Do you think your doctor rightly suggest you about referral? (if hospital facilities referred the patient)	1. a.Yes b.No; If No, please specify……………………………………….
Q.17	Do you know when a patient become sick, which one is the first centers you should go?	(Please write the response of the patients) ……………………………………………..
Q.18	Do you know, before going to any higher center, a referral note is required?	(Please write the response of the patients) ……………………………………………..
Q.19	Do you know any existing referral system in Bangladesh?	(Please write the response of the patients)
Q.20	Do you know, without a referral note, any tertiary care center can refuse your patients?	(Please write the response of the patients)
Q.21	Do you know, without a referral note, any tertiary care center can send back to you lower center?	(Please write the response of the patients)
Q.22	Distance of the hospital (km)	

**Table 5:** Bangla version of the questionnaire used in the study আপনার স্বাস্থ্য সম্পর্কিত প্রশ্ন করার আগে আমি কিছু প্রাথমিক তথ্য জানতে চাই। এই তথ্য সম্পূর্ণ গোপনীয় রাখা হবে এবং শুধুমাত্র গবেষণার উদ্দেশ্যে ব্যবহার করা হবে।

প্রশ্ন নং	প্রশ্ন	সম্ভাব্য উত্তর ও নির্দেশনা
Q.01	হাসপাতালের নাম কী?	______________________________
	বিভাগের নাম	অন্তবিভাগ/ বহির্বিভাগ
Q.02	পর্যবেক্ষণ অনুযায়ী লিঙ্গ লিপিবদ্ধ করুন	পুরুষ / মহিলা
Q.03	আপনার বয়স কত?	______________________________
Q.04	আপনার স্থায়ী ঠিকানা কী?	গ্রাম/এলাকা: __________ থানা/উপজেলা: __________ পৌরসভা/সিটি কর্পোরেশন: __________ জেলা: __________
Q.05	আপনার ধর্ম কী?	1. ইসলাম 2. হিন্দু 3. খ্রিস্টান 4. বৌদ্ধ 5. অন্যান্য (উল্লেখ করুন) __________
Q.06	আপনার বর্তমান পেশা কী?	1. সরকারি চাকরিজীবী 2. বেসরকারি চাকরিজীবী 3. স্বনিযুক্ত 4. নিয়োগকর্তা 5. বেতনভুক্ত কাজ করেন না (যদি না করেন: Q.208 উত্তর দিন)
Q.07	গত ১২ মাসে আপনার প্রধান পেশা কী ছিল?	1. আইনপ্রণেতা/ঊর্ধ্বতন কর্মকর্তা/ব্যবস্থাপক 2. পেশাজীবী 3. প্রযুক্তিবিদ/সহকারী পেশাজীবী 4. কেরানি 5. সেবা বা বিক্রয়কর্মী 6. কৃষি বা মৎস্যকর্মী 7. কারিগর বা দক্ষ শ্রমিক 8. প্ল্যান্ট/মেশিন অপারেটর বা সংযোজক 9. প্রাথমিক স্তরের কর্মী 10. সশস্ত্র বাহিনী
Q.08	আপনি বেতনভুক্ত কাজ করছেন না—এর প্রধান কারণ কী?	1. গৃহিণী/পরিবার পরিচর্যা 2. কাজ খুঁজেছেন কিন্তু পাননি 3. বিনা পারিশ্রমিকে কাজ/স্বেচ্ছাসেবামূলক কাজ 4. পড়াশোনা/প্রশিক্ষণ 5. অবসরপ্রাপ্ত/অতিবয়স্ক 6. অসুস্থতা 7. অন্যান্য (উল্লেখ করুন) __________
Q.09	আপনি সর্বোচ্চ কোন শিক্ষাগত যোগ্যতা সম্পন্ন করেছেন?	1. কোনো প্রাতিষ্ঠানিক শিক্ষা নেই 2. প্রাথমিকের নিচে 3. প্রাথমিক সম্পন্ন 4. মাধ্যমিক সম্পন্ন 5. উচ্চমাধ্যমিক সম্পন্ন 6. কলেজ/বিশ্ববিদ্যালয় সম্পন্ন 7. স্নাতকোত্তর ডিগ্রি
Q.10	রোগ	______________________________
	রেফারেল সিস্টেমের ব্যবহার (রেফারেলের উৎস)	______________________________
Q.11	আপনাকে এখানে (ভর্তি/ওপিডি) আসতে কে পরামর্শ দিয়েছেন?	1. নিজে (হ্যাঁ/না) 2. কবিরাজ (হ্যাঁ/না) 3. পল্লী ডাক্তার (হ্যাঁ/না) 4. বন্ধু/পরিবার (হ্যাঁ/না) 5. গ্রাম ডাক্তার (হ্যাঁ/না) 6. কমিউনিটি ক্লিনিক (হ্যাঁ/না) 7. ইউনিয়ন সাবসেন্টার (হ্যাঁ/না) 8. উপজেলা স্বাস্থ্য কমপ্লেক্স (হ্যাঁ/না) 9. জেলা হাসপাতাল (হ্যাঁ/না) 10. অন্য তৃতীয় স্তরের হাসপাতাল (হ্যাঁ/না; উল্লেখ করুন) 11. প্রাইভেট ক্লিনিক (হ্যাঁ/না) 12. অন্যান্য (উল্লেখ করুন)
Q.12	নিজ উদ্যোগে রেফারের কারণ	1. অপর্যাপ্ত চিকিৎসা (হ্যাঁ/না; উল্লেখ করুন) 2. অপর্যাপ্ত সুবিধা (হ্যাঁ/না; উল্লেখ করুন) 3. অনুপযুক্ত আচরণ (হ্যাঁ/না; উল্লেখ করুন) 4. বেশি খরচ (হ্যাঁ/না; উল্লেখ করুন) 5. অবস্থার অবনতি (হ্যাঁ/না; উল্লেখ করুন) 6. অন্যান্য (হ্যাঁ/না; উল্লেখ করুন)
Q.13	হাসপাতাল কর্তৃক রেফারের কারণ	1. জটিল কেস (হ্যাঁ/না; উল্লেখ করুন) 2. অতিরিক্ত ব্যবস্থাপনা প্রয়োজন (হ্যাঁ/না; উল্লেখ করুন) 3. অতিরিক্ত রোগ নির্ণয় যন্ত্র প্রয়োজন (হ্যাঁ/না; উল্লেখ করুন) 4. বিশেষজ্ঞ চিকিৎসকের অভাব (হ্যাঁ/না; উল্লেখ করুন) 5. উপযুক্ত সুবিধার অভাব (হ্যাঁ/না; উল্লেখ করুন) 6. রোগের সমাধান নেই/শেষ পর্যায় (হ্যাঁ/না; উল্লেখ করুন) 7. অন্যান্য (হ্যাঁ/না; উল্লেখ করুন)
Q.14	হাসপাতাল রেফার করলে, রোগী কি রেফারেল নোট পেয়েছেন?	হ্যাঁ / না (না হলে উল্লেখ করুন)
Q.15	এখানে ভর্তি হওয়া কি সঠিক সিদ্ধান্ত ছিল?	হ্যাঁ / না (হ্যাঁ হলে উল্লেখ করুন)
Q.16	ডাক্তার কি সঠিকভাবে রেফারেল পরামর্শ দিয়েছেন?	হ্যাঁ / না (না হলে উল্লেখ করুন)
Q.17	অসুস্থ হলে প্রথমে কোন কেন্দ্রে যাওয়া উচিত—আপনি জানেন?	রোগীর উত্তর লিখুন
Q.18	উচ্চতর কেন্দ্রে যাওয়ার আগে রেফারেল নোট প্রয়োজন—আপনি জানেন?	রোগীর উত্তর লিখুন
Q.19	বাংলাদেশে বিদ্যমান কোনো রেফারেল সিস্টেম সম্পর্কে জানেন?	রোগীর উত্তর লিখুন
Q.20	রেফারেল নোট ছাড়া তৃতীয় স্তরের হাসপাতাল রোগী গ্রহণ না-ও করতে পারে—আপনি জানেন?	রোগীর উত্তর লিখুন
Q.21	রেফারেল নোট ছাড়া তৃতীয় স্তরের হাসপাতাল রোগীকে নিম্নতর কেন্দ্রে ফেরত পাঠাতে পারে—আপনি জানেন?	রোগীর উত্তর লিখুন
Q.22	হাসপাতালের দূরত্ব (কিমি)	______________________________
